# Supplementary material for: The Systems Biology Research Tool: evolvable open-source software
Source: BMC Syst Biol. 2008 Jun 29;2:55. doi: 10.1186/1752-0509-2-55 (PMC2446383; doi:10.1186/1752-0509-2-55)
Supplement: Additional file 1 — SBRT Archive. An archive of the current version of the Systems Biology Research Tool. [file 1752-0509-2-55-S1.zip › sbrt-1.4.0/doc/developers_guide/api/sbrt/shell/mng/graph_theory/PathIdManager.html]

PathIdManager


|  |  |  |
| --- | --- | --- |
| **Overview** \| **Package** \| **Class** \| **Use** \| **Tree** \| **Deprecated** \| **Index** \| **Help** | |  |
| **PREV CLASS**   **NEXT CLASS** | **FRAMES**    **NO FRAMES**     **All Classes** |  |
| SUMMARY: NESTED \| FIELD \| CONSTR \| METHOD | DETAIL: FIELD \| CONSTR \| METHOD |  |


---


## sbrt.shell.mng.graph\_theory Class PathIdManager

```
java.lang.Object
  sbrt.shell.mng.AbstractProcessManager
      sbrt.shell.mng.graph_theory.PathIdManager
```

**All Implemented Interfaces:**: KernelProcessManager<java.lang.String,java.lang.String>, ProcessManager<java.lang.String,java.lang.String>

---

``` public final class PathIdManager extends AbstractProcessManager implements KernelProcessManager<java.lang.String,java.lang.String> ```

This class is used to manage simple path identification experiments.

**Author:**
:   This class was written and documented by Jeremiah Wright while
    in the Wagner lab.

---

| **Field Summary** | |
| --- | --- |
| `static java.lang.String` | `EDGE_FILE`             The keyword used to denote the input file containing a set of edges. |
| `static UnmodifiableSet<java.lang.String>` | `optionalKeywords`             The set of keywords understood, but not required, by instances of this class. |
| `static UnmodifiableSet<java.lang.String>` | `requiredKeywords`             The set of keywords required to be present in the `ManagerInput` objects supplied to instances of this class. |

**Fields inherited from class sbrt.shell.mng.AbstractProcessManager**

`ALGORITHM, COMPLETED, DATA_HEADERS, DEV_NULL, ELAPSED_TIME, FILE_FORMAT, INPUT_FILE, INPUT_FILE_FORMAT, INPUT_FILE_NAME_FILE, ITERATIONS, OUTPUT_FILE_FORMAT, OUTPUT_FILE_NAME, OUTPUT_FILE_NAME_FILE, PERCENT, PERCENT_COMPLETED, SEED, ZERO_CUTOFF`


**Constructor Summary**

`PathIdManager()`


| **Method Summary** | |
| --- | --- |
| `void` | `execute(java.io.PrintWriter out)`             Executes the process this process manager controls, and writes the status or results of this process to the provided print writer. |
| `static java.util.Set<DirectedGraph.Edge<java.lang.String>>` | `getEdges(java.lang.String fileName)`             Parses the file with the provided name, and returns the set of edges it contains. |
| `static <N> java.util.Set<DirectedGraph.Edge<N>>` | `getEdges(java.lang.String fileName, DirEdgeFormat<N> format)`             Parses the file with the provided name, and returns the set of edges it contains. |
| `static DirectedGraph<java.lang.String>` | `getGraph(ManagerInput<java.lang.String,java.lang.String> input, java.io.PrintWriter out)`             Opens the file denoted by `EDGE_FILE` in the provided manager input and returns the directed graph it contains. |
| `static DirectedGraph<java.lang.String>` | `getGraph(java.lang.String fileName)`             Parses the file with the provided name, and returns the directed graph it contains. |
| `static <N> DirectedGraph<N>` | `getGraph(java.lang.String fileName, DirEdgeFormat<N> format)`             Parses the file with the provided name, and returns the directed graph it contains. |
| `UnmodifiableSet<java.lang.String>` | `getOptionalKeys()`             Returns the set of keywords the provided `ManagerInput` object does not necessarily have to contain. |
| `PathIdProcess<java.lang.String>` | `getProcess()`             Returns the process this process manager controls. |
| `UnmodifiableSet<java.lang.String>` | `getRequiredKeys()`             Returns the set of keywords the provided `ManagerInput` object must contain. |
| `void` | `setInput(ManagerInput<java.lang.String,java.lang.String> input, java.io.PrintWriter out)`             Uses the provided input to prepare this process manager for execution. |

**Methods inherited from class sbrt.shell.mng.AbstractProcessManager**

`check, closeFile, closeRm, execute, getFileFormat, getFileFormat, getFileNameSource, getIterations, getSeed, getTextFileFormat, getTextFileFormat, getZeroCutoff, getZeroCutoff, parseInt, parseLong, printProgress, printProgress, printProgress, runProcess, setInput`

**Methods inherited from class java.lang.Object**

`clone, equals, finalize, getClass, hashCode, notify, notifyAll, toString, wait, wait, wait`

**Methods inherited from interface sbrt.shell.mng.ProcessManager**

`check, execute, setInput`

**Field Detail**

### EDGE\_FILE

```
public static final java.lang.String EDGE_FILE
```

:   The keyword used to denote the input file containing a set of edges.

    **See Also:**: Constant Field Values

---


### requiredKeywords

```
public static final UnmodifiableSet<java.lang.String> requiredKeywords
```

:   The set of keywords required to be present in the `ManagerInput`
    objects supplied to instances of this class.

---


### optionalKeywords

```
public static final UnmodifiableSet<java.lang.String> optionalKeywords
```

:   The set of keywords understood, but not required, by instances of this
    class.


**Constructor Detail**

### PathIdManager

```
public PathIdManager()
```


**Method Detail**

### getGraph

```
public static DirectedGraph<java.lang.String> getGraph(ManagerInput<java.lang.String,java.lang.String> input,
                                                       java.io.PrintWriter out)
```

:   Opens the file denoted by `EDGE_FILE` in the provided manager
    input and returns the directed graph it contains. The file must be a
    text file, and each non-commented line
    must be parsable with `DirEdgeFormatV1`.

    :   **Parameters:**: `input` - the input containing a value for `EDGE_FILE`.: `out` - the print writer to which a status message will be printed. **Returns:**: the directed graph contained in the file with the provided name.

---


### getEdges

```
public static java.util.Set<DirectedGraph.Edge<java.lang.String>> getEdges(java.lang.String fileName)
```

:   Parses the file with the provided name, and returns the set of edges it
    contains. The file must be a text-file, and each non-commented line
    must be parsable with `DirEdgeFormatV1`.

    :   **Parameters:**: `fileName` - the name of the file to be parsed. **Returns:**: the set of edges contained in the file with the provided name.

---


### getEdges

```
public static <N> java.util.Set<DirectedGraph.Edge<N>> getEdges(java.lang.String fileName,
                                                                DirEdgeFormat<N> format)
```

:   Parses the file with the provided name, and returns the set of edges it
    contains. The file must be a text-file, and each non-commented line
    must be parsable with the provided format.

    :   **Type Parameters:**: `N` - the node type. **Parameters:**: `fileName` - the name of the file to be parsed.: `format` - the format object used to parse the lines of the file. **Returns:**: the set of edges contained in the file with the provided name.

---


### getGraph

```
public static DirectedGraph<java.lang.String> getGraph(java.lang.String fileName)
```

:   Parses the file with the provided name, and returns the directed graph it
    contains. The file must be a text-file, and each non-commented line
    must be parsable with `DirEdgeFormatV1`.

    :   **Parameters:**: `fileName` - the name of the file to be parsed. **Returns:**: the directed graph contained in the file with the provided name.

---


### getGraph

```
public static <N> DirectedGraph<N> getGraph(java.lang.String fileName,
                                            DirEdgeFormat<N> format)
```

:   Parses the file with the provided name, and returns the directed graph it
    contains. The file must be a text-file, and each non-commented line
    must be parsable with the provided format.

    :   **Type Parameters:**: `N` - the node type. **Parameters:**: `fileName` - the name of the file to be parsed.: `format` - the format object used to parse the lines of the file. **Returns:**: the directed graph contained in the file with the provided name.

---


### getRequiredKeys

```
public UnmodifiableSet<java.lang.String> getRequiredKeys()
```

:   Returns the set of keywords the provided
    `ManagerInput` object must contain.

    :   **Specified by:**: `getRequiredKeys` in interface `ProcessManager<java.lang.String,java.lang.String>`
    :   **Returns:**: the set of keywords the provided `ManagerInput` object must contain.

---


### getOptionalKeys

```
public UnmodifiableSet<java.lang.String> getOptionalKeys()
```

:   Returns the set of keywords the provided
    `ManagerInput` object does not necessarily
    have to contain.

    :   **Specified by:**: `getOptionalKeys` in interface `ProcessManager<java.lang.String,java.lang.String>`
    :   **Returns:**: the set of keywords the provided `ManagerInput` object does not necessarily have to contain.

---


### getProcess

```
public PathIdProcess<java.lang.String> getProcess()
```

:   Returns the process this process manager controls.

    :   **Specified by:**: `getProcess` in interface `KernelProcessManager<java.lang.String,java.lang.String>`
    :   **Returns:**: the process this process manager controls. **Throws:**: `java.lang.IllegalStateException` - if no input was provided to this process manager via `AbstractProcessManager.setInput(ManagerInput)` or `setInput(ManagerInput, PrintWriter)`

---


### setInput

```
public void setInput(ManagerInput<java.lang.String,java.lang.String> input,
                     java.io.PrintWriter out)
```

:   Uses the provided input to prepare this process
    manager for execution. As the input is processed,
    informative messages are printed to the provided
    print writer.

    :   **Specified by:**: `setInput` in interface `ProcessManager<java.lang.String,java.lang.String>`
    :   **Parameters:**: `input` - all of the information required to prepare this process manager for execution.: `out` - the print writer to which all messages will be printed.

---


### execute

```
public void execute(java.io.PrintWriter out)
```

:   Executes the process this process manager controls,
    and writes the status or results of this process to the
    provided print writer.

    :   **Specified by:**: `execute` in interface `ProcessManager<java.lang.String,java.lang.String>`
    :   **Parameters:**: `out` - the print writer where status messages will be printed. **Throws:**: `java.lang.IllegalStateException` - if no input was provided to this process manager via `AbstractProcessManager.setInput(ManagerInput)` or `setInput(ManagerInput, PrintWriter)`


---


|  |  |  |
| --- | --- | --- |
| **Overview** \| **Package** \| **Class** \| **Use** \| **Tree** \| **Deprecated** \| **Index** \| **Help** | |  |
| **PREV CLASS**   **NEXT CLASS** | **FRAMES**    **NO FRAMES**     **All Classes** |  |
| SUMMARY: NESTED \| FIELD \| CONSTR \| METHOD | DETAIL: FIELD \| CONSTR \| METHOD |  |


---
